# Predicting prognosis of sepsis in patients based on right ventricular strain imaging development and validation of a nomogram model

**DOI:** 10.3389/fcvm.2025.1532674

**Published:** 2025-06-04

**Authors:** Qinxin Wang, Hongmin Chen, Bingyi Zhang, Chang Zhou, Boyuan Xing, Chang Li, Shijin Xu, Yun Liu

**Affiliations:** ^1^ Department of Ultrasound Imaging, the First College of Clinical Medical Science, China Three Gorges University & Yichang Central People’s Hospital, Yichang, China; ^2^ Department of General Practice, the First College of Clinical Medical Science, China Three Gorges University & Yichang Central People’s Hospital, Yichang, China

**Keywords:** sepsis, right ventricular systolic dysfunction, strain, nomogram model, prognostic analysis

## Abstract

**Background:**

The right ventricle (RV) plays a significant role in septic myocardial injury and associated organ dysfunction. Hence, identifying right ventricular systolic dysfunction (RVSD) early is crucial for improving outcomes in septic patients, yet current research on RVSD in sepsis remains limited.

**Objective:**

The study aims to identify risk factors for adverse outcomes in septic patients and construct a nomogram prediction model incorporating right ventricular strain and right ventricle–pulmonary artery coupling parameters.

**Methods:**

This single-center prospective study included 156 sepsis patients admitted from September 2021 to October 2024. General clinical, laboratory, and echocardiographic data were collected within 72 h of sepsis diagnosis. Prognosis was used to divide patients into two groups. Lasso regression was used to examine the baseline features of both groups. Multivariable logistic regression analysis and a nomogram were used to predict sepsis prognosis. The relationship between RVSD and 28-day mortality was examined.

**Results:**

Within 28 days, 52 of 141 sepsis patients died. Univariate analysis showed that the non-survivor cohort was older and had higher APACHE II and Sequential Organ Failure Assessment (SOFA) ratings and procalcitonin, B-type natriuretic peptide, cTnI, and lactate. RV-free wall strain (−18.9% ± 1.6% vs. −20.1% ± 1.5%, *p* < 0.001) and RV global strain (−18.6% ± 1.4% vs. −17.6% ± 1.0%, *p* < 0.001) were lower in the non-survivor group compared to the survivor cohort. PASP and RV-GS/PASP ratio significantly differed between the two groups (*p* < 0.05). Multivariable logistic regression analysis identified age >67 years, SOFA score ≥7.5, procalcitonin ≥5.7 ng/ml, lactate ≥3.5 mmol/L, RV-FWS ≥−19.4%, and RV-GS/PASP ≥−0.55 as independent risk factors for poor sepsis outcomes. The prognostic model using these six risk factors had an area under the curve (AUC) of 0.907 (95% CI: 0.858–0.954). Internal validation showed strong nomogram calibration with a C-index of 0.88.

**Conclusion:**

The RV-GS/PASP ratio demonstrated significant prognostic utility for predicting clinical outcomes in sepsis patients. Furthermore, the nomogram model incorporating age, SOFA score, procalcitonin, lactate, and RV-FWS exhibited excellent discriminative ability, with an AUC of 0.907.

## Introduction

Sepsis, a systemic inflammatory response syndrome triggered by infection, is often accompanied by severe organ dysfunction or failure. This complex condition arises when the body's response to an infection becomes dysregulated, leading to widespread inflammation that can affect multiple organ systems (1). Studies showed that complement activation products can trigger systemic inflammation, affecting organs such as the liver, lungs, and kidneys, ultimately resulting in multiple organ dysfunction syndrome and increased mortality (2). Among the pathophysiological studies conducted on cardiac dysfunction in sepsis, the left ventricle has been the primary focus of attention. However, as our understanding of the structure and function of the right ventricle (RV) continues to expand, its crucial role in the myocardial damage and the subsequent multi-organ dysfunction in sepsis becomes increasingly apparent. Research indicates that right ventricular systolic dysfunction (RVSD) affects nearly half of all patients diagnosed with sepsis and can result in a 40% mortality (3, 4). The findings of a study involving 393 patients in a critical care unit revealed that those who had RVSD had a 31% mortality after 28 days, whereas those who did not have RV dysfunction had a 16% mortality (5). Similarly, Innocenti et al. (6) conducted a study on 252 patients admitted to the emergency room and diagnosed with sepsis. They discovered that mortality at 28 days was 44% in patients who had RV dysfunction compared to 23% in their counterparts. The development of RVSD in sepsis might be caused by a number of causes (7), including the activation of pro-inflammatory cytokines, hypoxia, hypercapnia-induced vasoconstriction, pressure and volume overload, and myocardial ischemia. The timely recognition and prognostic evaluation of RVSD are essential for regulating fluid balance in patients with sepsis, executing protective breathing protocols, selecting and supervising the administration of inotropic medications, and ultimately preventing catastrophic right heart failure (HF) and multi-organ dysfunction.

Currently, there is no globally recognized definition of RVSD in sepsis. RV global strain (RV-GS) evaluated by speckle-tracking echocardiography (STE) is regarded as a dependable metric for assessing RV function (8). In contrast to traditional characteristics, RV-GS is less influenced by imaging angles. Moreover, RV-free wall strain (RV-FWS) diminishes reliance on left ventricular contraction. Despite the evidence indicating that strain imaging enhances the predictive assessment of RV function, its adoption is limited, and the ideal imaging method remains unclear. RV dysfunction occurs in approximately 30%–50% of patients with sepsis and septic shock, with its prevalence increasing to 72% when assessed by STE (9, 10).

Although RV-GS offers a quantitative assessment of myocardial function, it cannot consider the influence of afterload on RV performance. Recent research has indicated (11, 12) that evaluating right ventricular-pulmonary artery (RV-PA) coupling using the ratio of tricuspid annular plane systolic excursion to pulmonary artery systolic pressure (TAPSE/PASP) may reduce the impact of afterload, thereby enhancing the comprehension of RV function. Nevertheless, research investigating the correlation between RV-PA coupling and sepsis outcomes is limited. Furthermore, TAPSE solely indicates regional myocardial function, while RV-GS assesses overall RV function by monitoring myocardial tissue displacement. Consequently, would assessing RV-PA coupling by the RV-GS/PASP ratio serve as a more precise indicator of RVSD? Additionally, is there a correlation between RV-GS/PASP and prognosis in sepsis? To answer these questions, this study seeks to create a nomogram predictive model that integrates conventional echocardiographic parameters, RV-GS, RV-FWS, and RV-GS/PASP, while also examining the prognostic significance of RVSD, in patients with sepsis.

## Methods

### Study population

Adult sepsis patients treated at the Yichang Central People's Hospital between September 2021 and October 2024 were recruited. All patients were included according to the diagnostic criteria set by the 2016 International Sepsis Definitions Conference (13), requiring clinical evidence of infection and a Sequential Organ Failure Assessment (SOFA) score of ≥2 points. The exclusion criteria were left ventricular ejection fraction (LVEF) <50% or a history of acute myocardial infarction or HF within the past 3 months; severe arrhythmias (e.g., persistent or permanent atrial fibrillation) or patients who experienced cardiac arrest requiring cardiopulmonary resuscitation or defibrillation; history of congenital heart disease, moderate-to-severe valvular disease, or post-valve replacement surgery; pregnant or breastfeeding women; patients with chronic debilitating diseases; patients in the terminal stage of malignancy; unclear or incomplete imaging, or missing clinical data; and other conditions causing myocardial injury that leads to elevated cardiac enzymes or abnormal troponin levels. All patients or their legally authorized guardians were fully informed before examination and provided written informed consent. This study was approved by the Institutional Ethics Committee of the hospital (approval no.: PJ-KY2023-168-01).

### Clinical data

Height, weight, and body mass index (BMI) were measured within 72 h of hospital admission for all eligible participants. Medical history was obtained, and age, sex, heart rate, respiratory rate, temperature, and mean arterial pressure (MAP) were recorded. SOFA and Acute Physiology and Chronic Health Evaluation (APACHE) II scores were assessed for each sepsis patient. Venous blood samples were collected within 24 h of sepsis diagnosis using the ADVIA Centaur CP automated chemiluminescence system. Data on white blood cell count, hemoglobin, platelet count, albumin, creatinine, procalcitonin (PCT), cardiac troponin I (CTnI), B-type natriuretic peptide (BNP), and lactate (Lac) levels were collected. At least one sample was obtained after intensive care unit admission, with additional samples collected based on clinical requirements as determined by attending physicians. The worst values within 24 h of sepsis diagnosis were recorded. Additionally, data on the incidence of acute kidney injury (AKI) and acute respiratory distress syndrome, as well as the use of continuous renal replacement therapy (CRRT) and mechanical ventilation during the hospital stay, were collected. The primary clinical outcome was 28-day mortality.

### Echocardiography

The echocardiographic measurements were performed independently by two experienced sonographers. For each patient, cardiac function parameters were measured thrice consecutively, recording the average value. Routine echocardiography and two-dimensional STE (2D-STE) were conducted within 48 h of sepsis diagnosis using a GE Vivid E9 color Doppler ultrasound machine with an M5S 2D cardiac transducer (frequency range: 1.7–3.3 MHz). The scanning angle was maintained at <60°, and the frame rate was set between 60 and 90 frames per second. All participants were positioned with a synchronized left-sided precordial lead electrocardiography (ECG). The RV-focused apical four-chamber view was used to measure the RV end-diastolic diameter (RVDd), TAPSE and fractional area change (FAC). The TAPSE was assessed using M-mode echocardiography at the junction of the lateral tricuspid leaflet and the free wall of the RV. The FAC was calculated using the formula ([end-diastolic area−end-systolic area]/end-diastolic area) × 100%. All patients underwent PASP measurement within 48 h of sepsis diagnosis and prior to mechanical ventilation. The PASP was estimated based on the mean pressure gradient of tricuspid regurgitation using continuous-wave Doppler. Each parameter was measured three times consecutively, using the average value for analysis.

At least three cardiac cycles of 2D images were acquired, with the RV size maximized and the RV apex visualized throughout the entire cardiac cycle. The images were stored for offline analysis. Image analysis was conducted using the EchoPac workstation. The software automatically delineated the epicardial border and initiated tracking from the endocardial border at the level of the lateral tricuspid annulus, following the endocardial border to the level of the medial tricuspid annulus. The system generated longitudinal and circumferential strain values for different myocardial layers. The region of interest could be manually adjusted to ensure accurate tracking. The software calculated the RV-GS, which included both the free wall and interventricular septum, as well as the RV-FWS, defined as the mean strain of the three segments of the free wall. During image acquisition, patients were instructed to breathe steadily and, if necessary, hold their breath briefly to ensure optimal image quality.

### Statistical analysis

The data were analyzed utilizing SPSS version 26.0. Normality assessments were performed for continuous variables. Variables conforming to a normal distribution were represented as mean ± standard deviation (X ± s) and analyzed using the *t*-test. Non-normally distributed data were presented as median (interquartile range) [M (IQR)] and analyzed using the Wilcoxon rank-sum test. Categorical data were expressed as percentages (%) and analyzed using the chi-squared (χ²) test. Initially, univariate analysis was conducted, followed by the screening of important variables by LASSO regression utilizing ten-fold cross-validation. The ideal selection of variables was chosen, and receiver operating characteristic (ROC) curves were generated. The greatest Youden index was employed to ascertain the appropriate cutoff values, transforming the data into binary variables. Furthermore, logistic regression (LR) analysis was utilized to determine prognostic risk variables for sepsis. The risk factors were further analyzed using RStudio software, employing the “rms” function to develop a nomogram. Internal validation was conducted via the bootstrap approach, with the concordance index (C-index) computed to evaluate the discriminatory capacity of the model. Calibration curves and decision curves were generated to assess the consistency of the model. Survival analysis was performed utilizing Kaplan–Meier survival curves, and hazard ratios (HRs) were computed employing the Log-rank test. The threshold for statistical significance was established at *p* < 0.05.

## Results

### General characteristics

**Figure 1:**
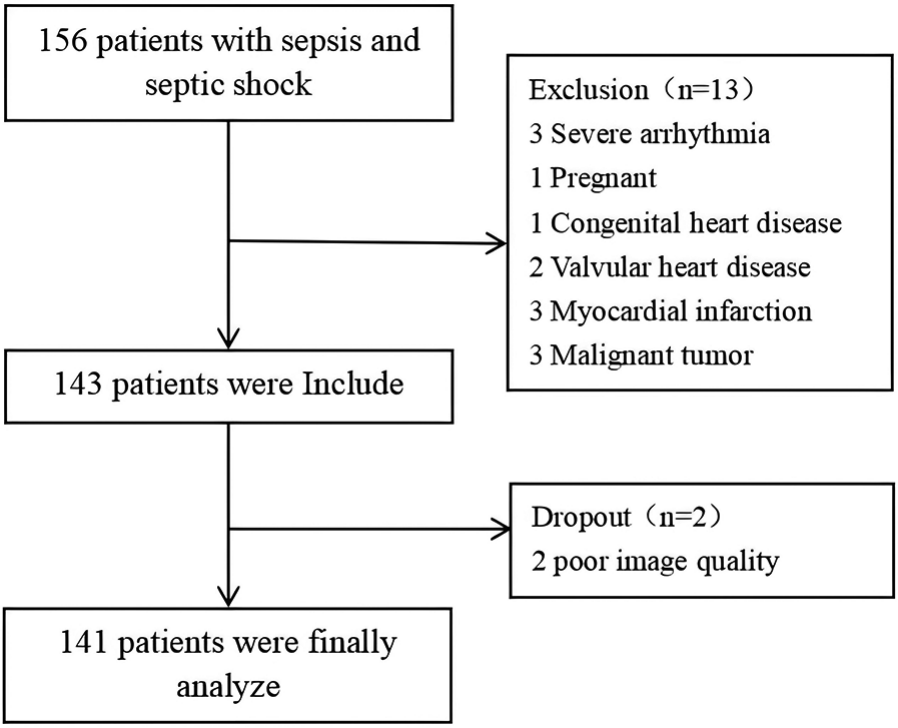
Enrollment process.

A total of 156 sepsis patients were initially screened, with 15 patients excluded according to predefined criteria: 3 cases with severe arrhythmia, 1 pregnant patient, 1 patient with congenital heart disease, 2 patients with valvular heart disease or post-valve replacement surgery, 3 patients with a history of myocardial infarction or myocarditis, 3 patients in the terminal stage of malignancy, and 2 patients with suboptimal image quality. A total of 141 patients participated in the trial, with males constituting 55.3% of the cohort (Figure 1).

Patients were divided into survivor and non-survivor groups based on their clinical outcomes. Baseline characteristics, including sex, BMI, the proportion of patients with shock, cardiovascular risk factors, or the distribution of infection sources, did not differ between the two groups (*p* > 0.05). Clinical indicators such as respiratory rate, MAP, heart rate, and body temperature also showed no significant differences between the two groups (*p* > 0.05). Laboratory indices, including white blood cell count, hemoglobin, albumin, platelet count, creatinine, and LDH, did not differ significantly between the groups (*p* > 0.05). Additionally, ultrasound parameters such as RVDd and FAC demonstrated no significant differences (Table 1).

**Table 1:** Baseline characteristics of patients in the survivors and non-survivors.

All patients (*n* = 141)		Survivors (*n* = 89)	Non-survivors (*n* = 52)	*P*-value
General characteristics				
Sex (male, %)	78 (55.3%)	45 (50.6%)	33 (63.5%)	0.137
Age (year)	65.6 ± 10.4	62.6 ± 10.3	70.7 ± 8.2	<0.001*
BMI (kg/m^2^)	23.9 ± 3.5	23.7 ± 3.5	24.2 ± 3.5	0.463
If shock (yes, %)	78 (55.3%)	48 (53.9%)	30 (57.7%)	0.665
Cardiovascular risk factors				
Hypertension (*n*/%)	63 (44.7%)	35 (39.3%)	28 (53.8%)	0.094
Diabetes (*n*/%)	32 (22.7%)	21 (23.6%)	11 (21.2%)	0.738
CHD (*n*/%)	25 (17.7%)	12 (13.5%)	13 (25.0%)	0.084
COPD (*n*/%)	22 (15.6%)	11 (12.4%)	11 (21.2%)	0.165
CRF (*n*/%)	23 (16.3%)	13 (14.6%)	10 (19.2%)	0.473
Clinical indicators				
Respiratory rate	21 (19, 23)	21 (20, 23)	21 (19, 24)	0.620
MAP (mmHg)	73.8 ± 14.1	72.1 ± 14.3	76.7 ± 13.2	0.061
Heart rate	102.8 ± 16.6	102.9 ± 15.9	102.7 ± 17.9	0.926
Temperature (°C)	38.0 (37.2, 38.9)	37.8 (37.2, 38.8)	38.0 (37.3, 39.0)	0.506
Organ function scoring				
APACHE II	19 (22, 28)	21 (19, 25)	27 (22, 31)	<0.001*
SOFA	8 (6, 11)	7 (5, 9)	9 (7, 13)	<0.001*
Laboratory indices				
WBC (10^9^/L)	7.3 (4.4, 13.3)	7.3 (4.3, 13.3)	7.5 (4.3, 11.8)	0.776
Hemoglobin (g/L)	98.5 ± 25.2	97.6 ± 25.3	100.0 ± 25.4	0.589
Platelet (10^9^/L)	99 (62, 173)	97 (62, 175)	101 (64, 171)	0.628
Albumin (g/L)	27.2 (23.5, 29.8)	26.4 (23.2, 29.7)	28.3 (24.8, 30.1)	0.185
Creatinine (umol/L)	122 (82, 236)	119 (80, 214)	125 (86, 254)	0.257
PCT (ng/ml)	7.3 (3.3, 36.4)	4.4 (1.9, 20.1)	15.0 (6.3, 55.0)	<0.001*
CTnI (ng/ml)	0.21 (0.05, 0.54)	0.12 (0.04, 0.45)	0.33 (0.19, 1.58)	<0.001*
BNP (pg/ml)	581 (224, 918)	430 (140, 678)	789 (490, 1,374)	<0.001*
Lac (mmol/L)	3.2 (2.2, 5.1)	2.8 (2.0, 4.4)	3.9 (2.2, 6.1)	0.016*
LDH (U/L)	304 (208, 434)	311 (218, 482)	303 (204, 415)	0.437
Ultrasound parameters				
RVDd (cm)	3.9 (3.7, 4.2)	3.9 (3.7, 4.2)	4.0 (3.7, 4.4)	0.507
TAPSE (cm)	1.7 (1.5, 1.8)	1.7 (1.5, 1.9)	1.5 (1.4, 1.8)	0.016*
FAC (%)	37 (35, 38)	37 (35, 38)	36 (34, 38)	0.128
RV-GS (%)	−18.2 ± 1.3	−18.6 ± 1.4	−17.6 ± 1.0	<0.001*
RV-FWS (%)	−19.7 ± 1.6	−20.1 ± 1.5	−18.9 ± 1.6	<0.001*
PASP (mmHg)	35 (31, 38)	33 (30, 37)	38 (35, 40)	0.001*
RV-GS/PASP	−0.53 ± 0.09	−0.57 ± 0.08	−0.46 ± 0.07	<0.001*
Infection sources				
Respiratory system (*n*/%)	49 (34.8%)	33 (37.1%)	16 (30.8%)	0.448
Digestive system (*n*/%)	42 (29.8%)	28 (31.5%)	14 (26.9%)	0.570
Urinary system (*n*/%)	22 (15.6%)	12 (13.5%)	10 (19.2%)	0.364
Superﬁcial tissue (*n*/%)	14 (9.9%)	6 (6.7%)	8 (15.4%)	0.098
Other system (*n*/%)	20 (14.2%)	13 (14.6%)	7 (13.5%)	0.851

BMI, body mass index; CHD, coronary heart disease; COPD, chronic obstructive pulmonary disease; CRF, chronic renal failure; MAP, mean arterial pressure; APACHE II Score, acute physiology and chronic health evaluation II score; SOFA Score, sequential organ failure assessment score; WBC, white blood cell; PCT, procalcitonin; CTnI, cardiac troponin I; BNP, brain natriuretic peptide; Lac, lactate; LDH, lactate dehydrogenase; RVDd, right ventricular end-diastolic diameter; TAPSE, tricuspid annular plane systolic excursion; FAC, fractional area change of the right ventricle; RV-GS, right ventricular global strain; RV-FWS, right ventricular free wall strain; PASP, pulmonary artery systolic pressure.

* Indicates *p* < 0.05.

Compared to the survivor group, non-survivors were older and had significantly higher APACHE II and SOFA scores. Biomarker levels, including PCT, BNP, CTnI, and Lac, were elevated in non-survivors. The absolute values of RV-FWS (−18.9% ± 1.6% vs. −20.1% ± 1.5%, *p* < 0.001), RV-GS (−17.6% ± 1.0% vs. −18.6% ± 1.4%, *p* < 0.001), and the RV-GS/PASP ratio (−0.46 ± 0.07 vs. −0.57 ± 0.08, *p* < 0.001) were significantly lower in non-survivors. Both TAPSE and PASP showed statistically significant differences between the two groups (*p* < 0.05) (Table 1).

### LASSO regression feature selection

**Figure 2:**
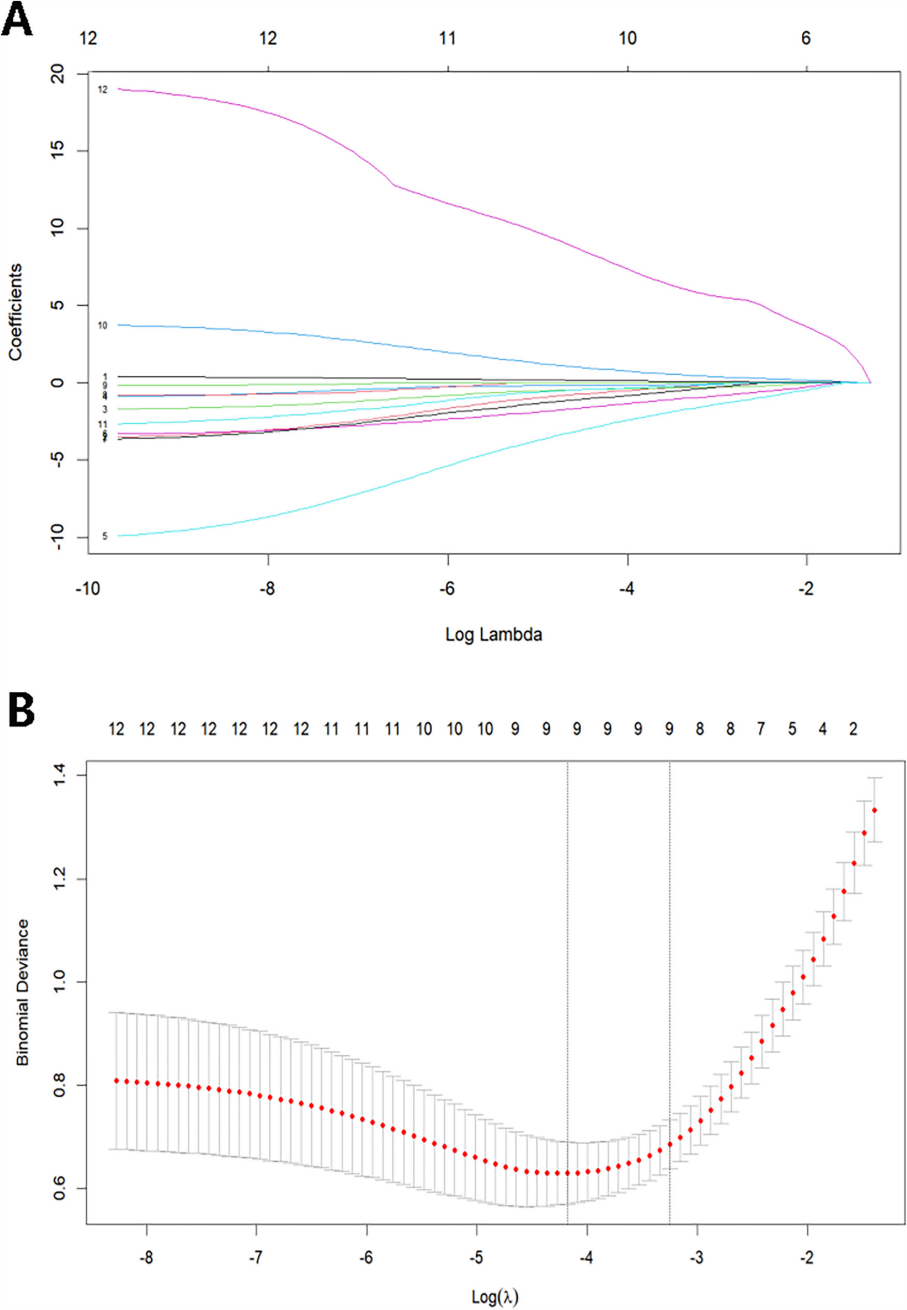
**(A)** LASSO coefficient profiles of the prognosis variables. **(B)** Selection of optimal lambda value using ten-fold cross-validation by the minimum criteria.

Thirty fundamental clinical signs and 7 auxiliary examination indicators for septic patients were used in this investigation. LASSO regression was utilized to identify predictive factors for worse outcomes in sepsis due to the vast number of variables while minimizing multicollinearity and avoiding model overfitting. The dependent variable was adverse outcomes, and the independent factors were the 12 variables that showed significant differences in the univariate analysis. Then, a ten-fold cross-validation was performed to choose the best model. Every curve in Figure 2A depicts the course of the coefficient change of a variable. Figure 2B demonstrates the correlation between the log-transformed λ (lambda) and the binomial deviation. The log λ with the minimum mean error, lambda.min, and the log λ with one standard error, lambda.1se, are shown by the two vertical dashed lines. We chose the characteristics using lambda.1se to produce a streamlined and effective model. The best predictors were 9 non-zero coefficient variables: age, PCT, Lac, RV-GS, RV-FWS, PASP, SOFA score, APACHE II score, and RV-GS/PASP.

We subsequently calculated variance inflation factors (VIFs) for the selected variables for multicollinearity assessment to mitigate multicollinearity among variables and prevent model overfitting. All continuous variables were standardized using Z-score normalization before computing VIF values through linear regression modeling. The threshold was set at VIF <5 to indicate an acceptable level of collinearity. All obtained VIF values were <5, demonstrating satisfactory independence among the final model variables (Table 2).

**Table 2:** Variance inflation factors analysis of predictor variables.

Variable	VIF	Tolerance	Collinearity diagnosis
Age	1.07	0.94	None
SOFA	1.21	0.83	None
APACHE II	1.44	0.70	None
PCT	1.14	0.88	None
Lac	1.15	0.87	None
RV-GS	2.01	0.50	None
RV-FWS	1.22	0.82	None
PASP	1.07	0.94	None
RV-GS/PASP	2.28	0.44	None

VIF, variance inflation factor.

### ROC curves and logistic regression analysis

**Figure 3:**
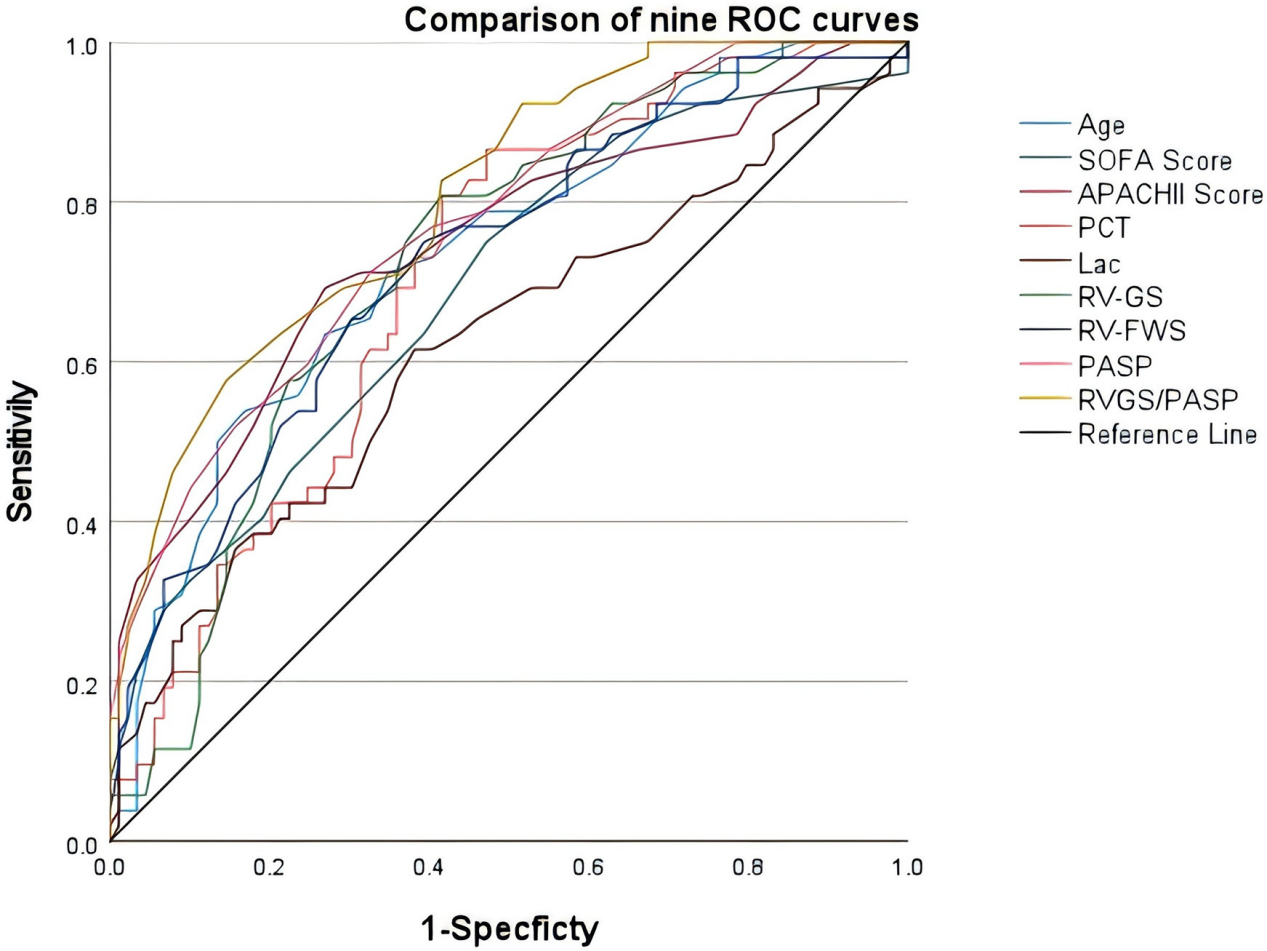
Receiver operating characteristic curves of age, SOFA score, APACHE II score, PCT, Lac, RV-GS, RV-FWS, PASP, and RV-GS/PASP in evaluating the prognosis of sepsis patients.

ROC curves were constructed to predict the prognosis of sepsis patients based on the 9 variables selected by LASSO regression, namely age, SOFA score, APACHE II score, PCT, Lac, RV-GS, RV-FWS, PASP, and the RV-GS/PASP ratio (Figure 3). The data were transformed into binary variables utilizing the greatest Youden index (Table 3).

**Table 3:** Receiver operating characteristic curve analysis for the prognosis evaluation of sepsis patients.

Parameters	AUC	Cut-off value	Sensitivity (%)	Specificity (%)	95% CI		Youden index	*P*-value
					Lower	Upper		
Age	0.690	67	71.2	65.2	0.651	0.820	0.440	<0.001
SOFA	0.807	7.5	83.7	54.9	0.671	0.843	0.386	<0.001
APACHE II	0.745	24.5	69.2	73.0	0.675	0.868	0.422	<0.001
PCT	0.708	5.7	80.8	58.4	0.615	0.844	0.403	0.003
Lac	0.622	3.5	61.5	61.8	0.522	0.721	0.233	0.016
RV-GS	0.725	−18.9	80.8	58.4	0.641	0.809	0.392	<0.001
RV-FWS	0.770	−19.4	67.3	69.7	0.692	0.849	0.370	<0.001
PASP	0.727	35.5	71.2	67.4	0.641	0.813	0.386	<0.001
RV-GS/PASP	0.803	−0.55	82.7	58.4	0.731	0.875	0.411	<0.001

AUC, area under the curve; 95% CI, 95% confidence interval.

The 9 binary variables were included in a multivariate logistic regression analysis using a stepwise forward selection method, with variables having *p* > 0.1 sequentially removed. Ultimately, six factors were identified as independent risk factors for poor prognosis in sepsis: age ≥67 years, SOFA score ≥7.5, PCT ≥5.7 ng/ml, Lac ≥3.5 mmol/L, RV-FWS ≤−19.4%, and RV-GS/PASP ≥−0.55 (Table 4).

**Table 4:** Multivariate logistic regression analysis.

Parameters	Coef.	SE	Wald x^2^	*P*-value	OR	95% CI	
						Lower	Upper
Age ≥67 years	2.449	0.564	18.869	<0.001	11.578	3.835	34.959
SOFA score ≥7.5	1.086	0.524	4.293	0.038	2.963	1.06	8.277
PCT ≥5.7 ng/ml	2.268	0.550	16.989	<0.001	9.656	3.285	28.385
Lac ≥3.5 mmol/L	1.287	0.512	6.332	0.012	3.623	1.329	9.874
RV-FWS ≥−19.4%	0.986	0.509	3.751	0.053	2.680	0.988	7.267
RV-GS/PASP ≥−0.55	1.796	0.557	10.4	0.001	6.026	2.023	17.95

Coef., coefficient of regression; SE, standard error; Wald x2, Wald Chi-square value; OR, odds ratio.

### The construction and validation of the model

**Figure 4:**
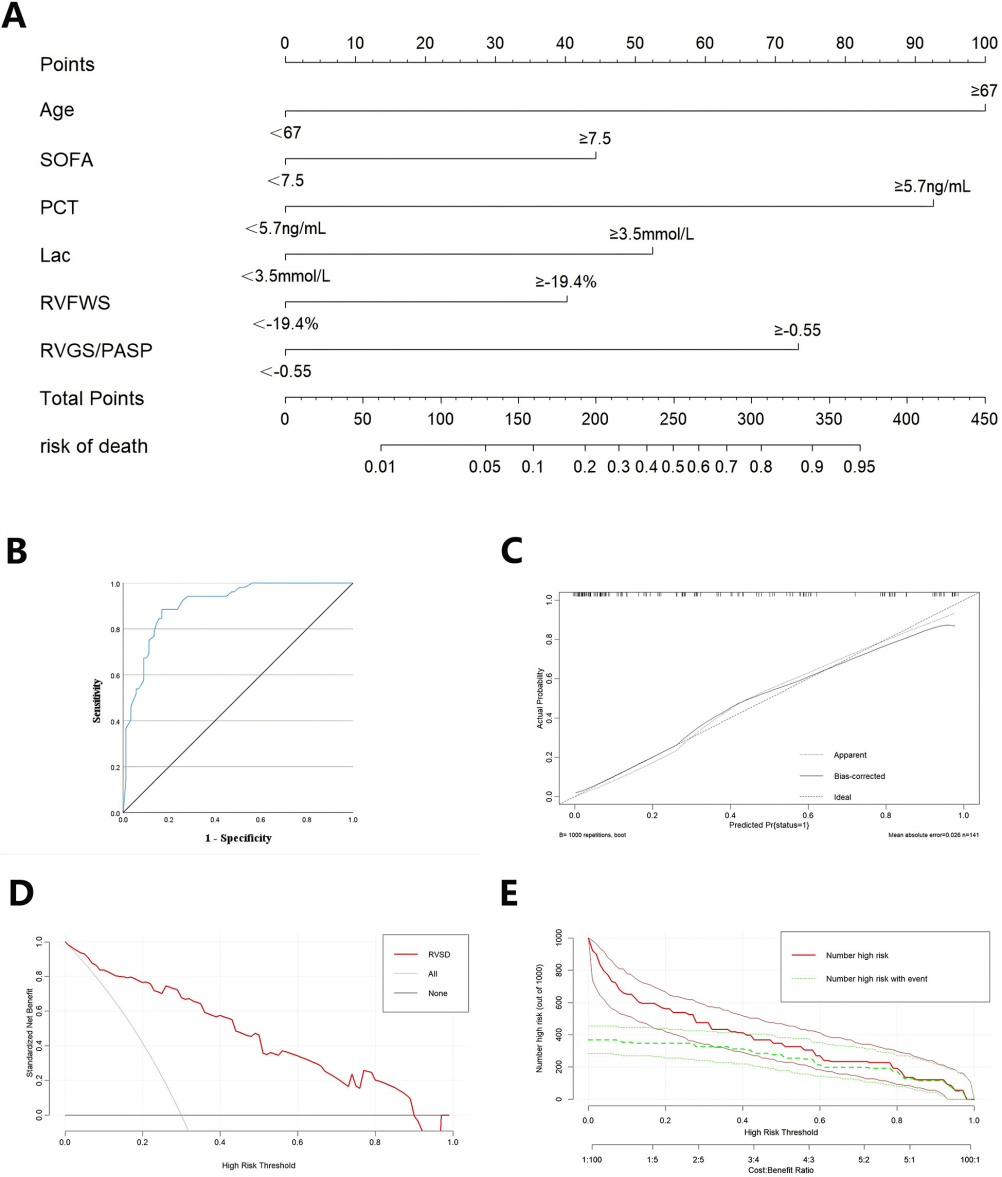
The validation of combined nomogram. **(A)** A combined nomogram model based on clinicopathological features and ultrasonographic characteristics. **(B)** ROC curve analysis of the nomogram model. **(C)** The calibration curve of the nomogram model. **(D)** Decision curves of the nomogram model. **(E)** Clinical impact curves of the nomogram model.

The 6 variables (age ≥67 years, SOFA score ≥7.5, PCT ≥5.7 ng/ml, Lac ≥3.5 mmol/L, RV-FWS ≥−19.4%, and RV-GS/PASP ≥−0.55) were included in an LR model. The goodness of fit of the model was assessed using the Hosmer-Lemeshow test, with the result indicating good model fit (*p* = 0.864). The predictive performance of the model for sepsis-induced cardiac dysfunction was evaluated using ROC analysis, demonstrating an area under the curve (AUC) of 0.907 (*p* < 0.001; Figure 4B).

A nomogram model was developed in RStudio to concurrently assess the 6 characteristics linked to unfavorable prognosis in sepsis patients based on the findings of the LR analysis (Figure 4A). The nomogram underwent internal validation through the bootstrap resampling technique, and calibration and decision curve analysis (DCA) plots were produced. The calibration curve demonstrated strong concordance with the ideal reference line, signifying satisfactory calibration of the model (Figure 4C). Upon internal validation, the model attained a C-index of 0.88, indicating adequate discriminative capability. The DCA revealed that the red curve did not intersect the gray diagonal line and predominantly stayed above the black horizontal line, signifying a favorable net benefit of the model (Figure 4D). The clinical impact curve demonstrated that when the threshold probability exceeded 0.6, the predictions of the model closely aligned with the actual outcomes, signifying high clinical predictive efficacy (Figure 4E).

### Prognostic analysis

**Figure 5:**
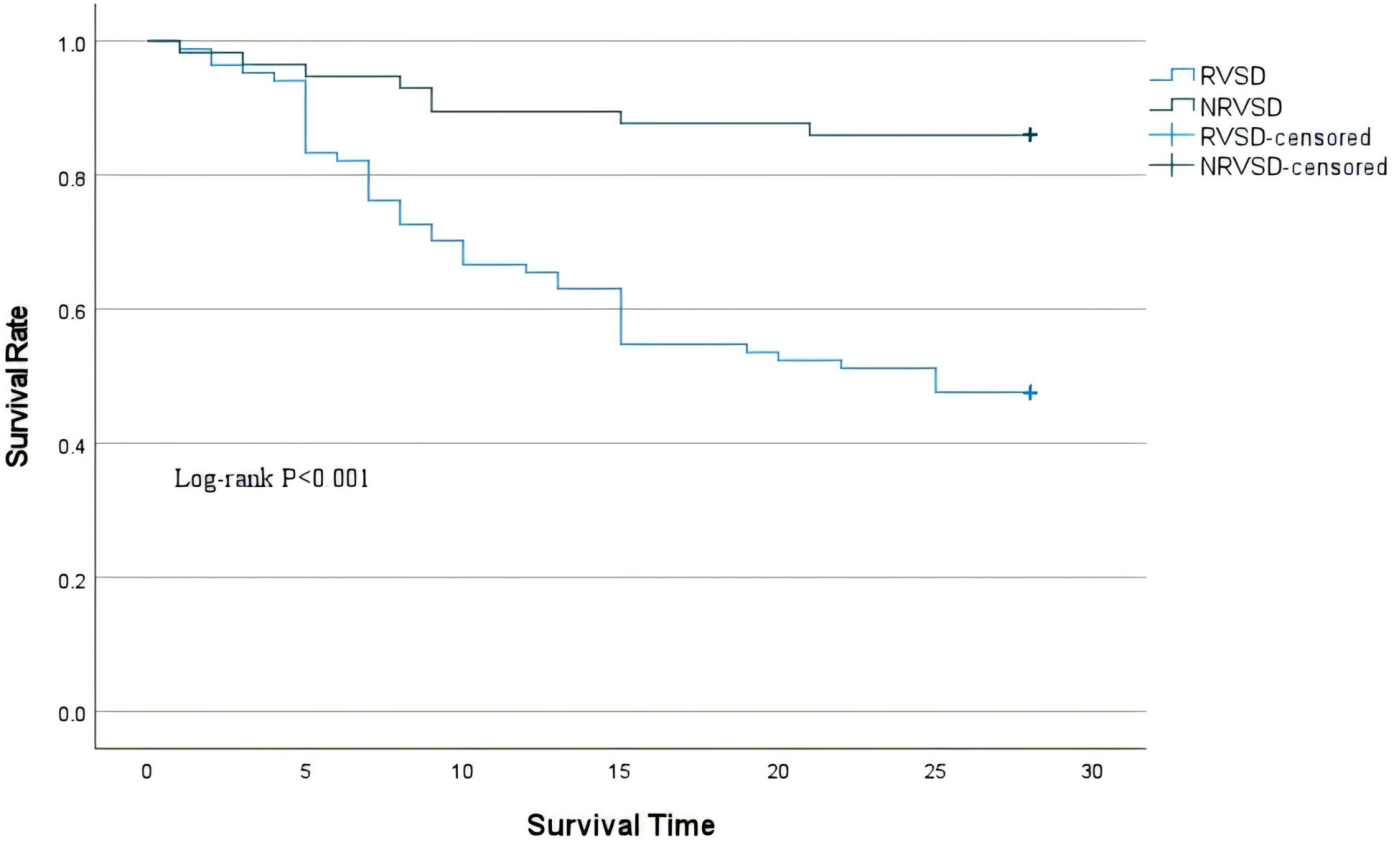
Kaplan–Meier survival curves for 28-day mortality of the two groups.

Patients were categorized into non-RVSD and RVSD groups according to the threshold value of RV-GS/PASP at ≥−0.55. The RVSD group had a markedly elevated mortality compared to the non-RVSD group (63.4% vs. 25.4%), along with a greater AKI frequency. The RVSD group had a higher requirement for vasopressor therapy (48.8% vs. 28.8%), mechanical ventilation (61.0% vs. 44.1%), and CRRT (53.7% vs. 35.6%) (Table 5). Survival analysis revealed that the 28-day mortality risk was markedly elevated in the RVSD group compared to the non-RVSD group (Figure 5).

**Table 5:** Comparison analysis of prognosis and complications between the two groups.

Variable	Non-RVSD	RVSD	*P*-value
ICU mortality (*n*/%)	15 (25.4%)	52 (63.4%)	<0.001*
AKI incidence (*n*/%)	17 (28.8%)	38 (46.3%)	0.035*
ARDS incidence (*n*/%)	16 (27.1%)	31 (37.8%)	0.184
MV rate (*n*/%)	26 (44.1%)	50 (61.0%)	0.047*
Vasopressor usage (*n*/%)	17 (28.8%)	40 (48.8%)	0.017*
CRRT rate (*n*/%)	21 (35.6%)	44 (53.7%)	0.034*

ARDS, acute respiratory distress syndrome; MV, mechanical ventilation.

*P < 0.05.

## Discussion

The assessment of RV function remains a significant challenge in clinical cardiology due to its unique anatomical structure and complex motion patterns. The RV, characterized by a crescent-shaped geometry, prominent trabeculations, thin walls, and intricate contraction mechanics, is markedly influenced by left ventricular (LV) mechanical coupling and intrathoracic pressure variations (14). These morphological and physiological particularities confer inherent limitations to conventional echocardiographic techniques for RV evaluation. Despite offering operational simplicity, widely used parameters such as TAPSE and FAC provide a unidimensional assessment with notable measurement constraints. Furthermore, these conventional indices primarily reflect global alteration in systolic function, limited sensitivity for early myocardial injury, and restricted prognostic value.

The advent of STE has introduced transformative solutions to these limitations. STE quantifies three-dimensional myocardial deformation without geometric assumptions or angle dependency by tracking spatial displacement of natural acoustic markers within myocardial tissue, achieving superior reproducibility and accuracy. Substantial clinical evidence confirms that strain parameters can detect subclinical dysfunction before overt structural remodeling (15, 16), providing critical windows for timely intervention. The 2023 ESC guidelines formally incorporated RV strain analysis as a core parameter for RV assessment, thereby highlighting its unique prognostic value in cardiovascular diseases. Meta-analyses cited in the guidelines (17) established RV-FWS as a robust predictor of cardiovascular events and all-cause mortality in pulmonary hypertension and demonstrated its superior prognostic performance over TAPSE and FAC in HF patients. Additionally, a comprehensive assessment of LV, RV, and left atrial strains significantly enhances long-term survival prediction in acute myocardial infarction. From myocardial biomechanical and pathophysiological perspectives, the distinctive sensitivity of RV strain stems from its quantification of longitudinal myocardial fiber deformation. As longitudinal fibers predominantly reside in the endocardial layer — the layer most vulnerable to the impairment in oxygen delivery and coronary blood flow — longitudinal strain exhibits heightened sensitivity for early dysfunction (18). Consequently, longitudinal strain abnormalities typically develop earlier than other indicators during initial myocardial injury, explaining its superior capability for early RV dysfunction detection.

This study employed a forward stepwise multivariate LR to identify independent risk factors for adverse outcomes in sepsis patients. The final model identified 6 statistically significant predictors (*p* < 0.05): age ≥72 years [odds ratio (OR) = 11.578, 95% confidence interval (CI) 3.835–34.959, *p* < 0.001] emerged as the strongest predictor, underscoring advanced age as a critical risk factor. SOFA score ≥9.5 points (OR = 2.963, 95% CI 1.016–8.277, *p* = 0.038) reflected a significant association between multi-organ dysfunction severity and poor outcomes. Among metabolic indicators, PCT ≥4.8 ng/ml (OR = 9.656, 95% CI 3.285–28.385, *p* < 0.001) and Lac ≥3.5 mmol/L (OR = 3.623, 95% CI 1.329–9.874, *p* = 0.012) indicated that worsening systemic inflammation and hypoperfusion increase the mortality risk. Notably, RV functional parameters demonstrated exceptional predictive capacity: RV-FWS ≥−19.4% (OR = 2.680, 95% CI 0.988–7.267, *p* = 0.043) and RV-GS/PASP ≥−0.55 (OR = 6.026, 95% CI 2.023–17.945, *p* = 0.001) both confirmed RV dysfunction as an independent prognostic determinant in sepsis.

Univariate analysis revealed significant correlations between RV-GS, RV-FWS, and adverse clinical outcomes in sepsis patients (*p* < 0.001). However, multivariate LR demonstrated that only RV-FWS remained an independent predictor of 28-day mortality (OR = 2.680, 95% CI 0.988–7.267) after adjusting for clinical confounders, including age, SOFA score, and Lac levels. This finding aligns with the established prognostic value of RV-FWS in populations with other cardiac dysfunction. Carluccio et al. (19) conducted a comparative analysis of left and right ventricular strain parameters in HF with reduced ejection fraction patients, accounting for LV function. They found that RV-FWS (HR = 1.82, 95% CI 1.45–2.29) demonstrated superior predictive power for all-cause mortality compared to RV-GS (HR = 1.60, 95% CI 1.29–1.99), maintaining significant independence even after adjusting for LV function (*p* < 0.001). Chen et al. (20) reported that RV-FWS (HR = 3.97, 95% CI 1.85–8.51) could more sensitively detect early RV systolic dysfunction than conventional parameters, and sepsis patients with reduced absolute RV-FWS values had lower 30-day survival, suggesting that strain parameters can identify subclinical myocardial injury undetectable by traditional methods, particularly revealing concealed RV contractile reserve depletion during LV compensation. de Braga et al. (21) further validated the prognostic value of RV strain, demonstrating that RV-GS at admission predicts in-hospital mortality. Notably, sepsis survivors exhibited significantly improved RV-FWS following treatment compared to non-survivors. Hence, RV-FWS serves not only as a baseline risk stratification tool but may also facilitate dynamic monitoring of therapeutic response and disease progression. In summary, this study not only underscores the prognostic value of RV-FWS in sepsis patients but also elucidates the pivotal role of RV decompensation in sepsis-induced multi-organ dysfunction. Additionally, these findings provide critical evidence for refining risk stratification and advancing precision management strategies in sepsis care.

The concept of “RV-pulmonary artery coupling” evaluates the matching between RV function and circulation. Physiologically, ventricular and vascular elasticity maintain optimal coupling through dynamic regulation, with pathological changes disrupting this balance and leading to RV failure (22). With the progression of septic shock severity, the protective RV-PA coupling mechanism fails, resulting in decoupling. Given the afterload-dependence of RV function, a combined assessment of RV function and pulmonary circulation may better predict outcomes. Zhang et al. (23) conducted a 1-year follow-up of 118 septic shock patients, finding that non-survivors had lower TAPSE, higher PASP, and reduced TAPSE/PASP ratio, with TAPSE/PASP ≤0.5 mm/mmHg significantly predicting 1-year mortality. This reflects impaired RV compensatory capacity against increased afterload. Bowcock et al. (24) further established the inverse relationship of the TAPSE/tricuspid regurgitant velocity ratio with mortality (HR = 0.927, 95% CI 0.872–0.985, *p* < 0.05), with higher ratios (better RV-PA coupling) independently correlating with lower mortality risk. As a TAPSE/PASP alternative, RV-GS/PASP more precisely evaluates RV contractility-afterload matching. RV-GS/PASP demonstrated superior 28-day mortality prediction at a cutoff of −0.55 compared to RV-GS alone (*p* < 0.001), suggesting its dual utility for early RV-PA decoupling detection and a warning indicator for RV decompensation in sepsis.

Furthermore, patients categorized into the RVSD group based on the RV-GS/PASP ratio cutoff of −0.55 demonstrated significantly worse prognostic characteristics. Specifically, they had a markedly higher incidence of AKI (46.3% vs. 28.8%, *p* < 0.001), likely mechanistically linked to renal venous congestion and decreased renal perfusion pressure secondary to RV hypertension (25). Additionally, the increased requirement for vasoactive agents (48.8% vs. 28.8%, *p* = 0.017) suggested more severe vascular paralysis in RVSD patients. The elevated demand for CRRT (53.7% vs. 35.6%, *p* = 0.034) not only reflected AKI severity but also correlated strongly with reduced fluid tolerance in right heart dysfunction (26). Most critically, the RVSD group exhibited significantly higher 28-day all-cause mortality (63.4% vs. 25.4%, *p* < 0.001), potentially indicating a dose-dependent relationship between myocardial mechanical deterioration and outcomes. The underlying mechanisms involve decreased RV energy metabolic efficiency from RV-PA uncoupling and LV filling restriction due to ventricular interdependence, which collectively exacerbate systemic hypoperfusion and amplify the inflammatory cascade, ultimately driving irreversible multi-organ failure (27). These findings confirm that the RV-GS/PASP ratio ≤−0.55 serves not only as a sensitive prognostic marker but also carries direct value for clinical intervention guidance.

Despite numerous prognostic models for sepsis outcomes, they have limitations. Some models rely solely on clinical indicators; for instance, one study constructed a 28-day mortality prediction model using the peripheral perfusion index (PPI) (28), yet single parameters cannot comprehensively capture the complex pathophysiology of sepsis. Machine learning approaches, such as Gradient Boosting Decision Tree algorithms, demonstrated high performance in predicting sepsis mortality by analyzing large clinical datasets (29), but their “black-box” nature limits clinical interpretability. Other biomarker-based models incorporating serum markers with clinical features (30) still show constrained predictive capability. Consequently, this study innovatively developed the first nomogram model incorporating RV strain parameters (RV-FWS and RV-GS/PASP) for sepsis prognosis, providing a novel quantitative assessment tool. Compared to conventional unidimensional metrics such as TAPSE, this multidimensional model better reflects the severity of RV contractile impairment. RV-GS/PASP showed the highest contribution weight (OR = 1.32, 95% CI:1.15–1.51), outperforming isolated RV-GS or PASP measurements by eliminating confounding from pure pulmonary hypertension or myocardial suppression, thus more accurately quantifying RV afterload adaptation capacity. This supports the evaluation of both contractility and afterload status in sepsis-induced RV dysfunction. This study adhered to the TRIPOD type 2a guidelines (development with internal validation), with subsequent external validation to be performed according to type 3 protocols (independent cohort validation). Upon successful validation, the next phase will involve designing a randomized controlled trial to evaluate whether early right heart function–guided therapy (e.g., optimized fluid management or pulmonary vasodilator administration) based on this model can improve clinical outcomes. Future research may explore artificial intelligence–assisted automated strain analysis to reduce operator dependence and enhance the applicability of the model in primary care hospitals.

This study has several limitations. First, the single-center design and relatively small sample size may limit the generalizability of the findings. Although we mitigated overfitting risks through stringent inclusion criteria and bootstrap internal validation, the performance of the model in patients with chronic cardiopulmonary comorbidities or diverse healthcare resource settings requires external validation. Second, while our RV-FWS cutoff (−19.4%) aligns with the range reported in previous sepsis studies (−18% to −21%) (31), the observed variations might have stemmed from differences in ultrasound acquisition protocols or patient severity stratification. Notably, compared to large multicenter studies (e.g., PROGRESS), this study introduces RV-GS/PASP as a novel composite index that demonstrates superior discriminative power over conventional hemodynamic parameters (e.g., TAPSE, FAC), offering a new perspective for bedside assessment of RV dysfunction. Despite the promise of strain imaging in septic cardiomyopathy, reproducibility remains limited by acquisition variability. Our data revealed significant inter-observer strain discrepancies (15%, *p* < 0.05), most probably attributable to inconsistent probe pressure. Therefore, automation may mitigate this operator-dependent bias.

## Conclusion

The RV-GS/PASP ratio demonstrated significant prognostic utility for predicting clinical outcomes in sepsis patients. Furthermore, the nomogram model incorporating age, SOFA score, PCT, Lac, and RV-FWS exhibited excellent discriminative ability, with an AUC of 0.907 and a C-index of 0.887.

## Data Availability

The datasets presented in this article are not readily available because the data is securely stored in Yichang Central Peoples Hospital under restricted access to protect participant confidentiality. Requests to access the datasets should be directed to the corresponding author.
